# Improved trends in survival and engraftment after single cord blood transplantation for adult acute myeloid leukemia

**DOI:** 10.1038/s41408-022-00678-6

**Published:** 2022-05-25

**Authors:** Takaaki Konuma, Shohei Mizuno, Tadakazu Kondo, Yasuyuki Arai, Naoyuki Uchida, Satoshi Takahashi, Masatsugu Tanaka, Takuro Kuriyama, Shigesaburo Miyakoshi, Makoto Onizuka, Shuichi Ota, Yasuhiro Sugio, Yasushi Kouzai, Toshiro Kawakita, Hikaru Kobayashi, Yukiyasu Ozawa, Takafumi Kimura, Tatsuo Ichinohe, Yoshiko Atsuta, Masamitsu Yanada, for the Adult Acute Myeloid Leukemia Working Group of the Japanese Society for Transplantation and Cellular Therapy

**Affiliations:** 1 Department of Hematology/Oncology, The Institute of Medical Science, The University of Tokyo, Tokyo, Japan; 2 Division of Hematology, Department of Internal Medicine, Aichi Medical University, Nagakute, Japan; 3 Department of Hematology and Oncology, Graduate School of Medicine, Kyoto University, Kyoto, Japan; 4 Department of Hematology, Toranomon Hospital, Tokyo, Japan; 5 Division of Clinical Precision Research Platform, The Institute of Medical Science, The University of Tokyo, Tokyo, Japan; 6 Department of Hematology, Kanagawa Cancer Center, Yokohama, Japan; 7 Department of Hematology, Hamanomachi Hospital, Fukuoka, Japan; 8 Department of Hematology, Tokyo Metropolitan Geriatric Hospital, Tokyo, Japan; 9 Department of Hematology and Oncology, Tokai University School of Medicine, Isehara, Japan; 10 Department of Hematology, Sapporo Hokuyu Hospital, Sapporo, Japan; 11 Department of Internal Medicine, Kitakyushu City Hospital Organization, Kitakyushu Municipal Medical Center, Kitakyushu, Japan; 12 Department of Transfusion Medicine, Tokyo Metropolitan Tama Medical Center, Tokyo, Japan; 13 Department of Hematology, National Hospital Organisation Kumamoto Medical Center, Kumamoto, Japan; 14 Department of Hematology, Nagano Red Cross Hospital, Nagano, Japan; 15 Department of Hematology, Japanese Red Cross Nagoya First Hospital, Nagoya, Japan; 16 Preparation Department, Japanese Red Cross Kinki Block Blood Center, Osaka, Japan; 17 Department of Hematology and Oncology, Research Institute for Radiation Biology and Medicine, Hiroshima University, Hiroshima, Japan; 18 Japanese Data Center for Hematopoietic Cell Transplantation, Nagoya, Japan; 19 Department of Registry Science for Transplant and Cellular Therapy, Aichi Medical University School of Medicine, Nagakute, Japan; 20 Department of Hematology and Cell Therapy, Aichi Cancer Center, Nagoya, Japan

**Keywords:** Acute myeloid leukaemia, Cancer immunotherapy

## Abstract

Unrelated cord blood transplantation (CBT) is an alternative curative option for adult patients with acute myeloid leukemia (AML) who need allogeneic hematopoietic cell transplantation (HCT) but lack an HLA-matched related or unrelated donor. However, large-scale data are lacking on CBT outcomes for unselected adult AML. To investigate the trends of survival and engraftment after CBT over the past 22 years, we retrospectively evaluated the data of patients with AML in Japan according to the time period of CBT (1998–2007 vs 2008–2013 vs 2014–2019). A total of 5504 patients who received single-unit CBT as first allogeneic HCT for AML were included. Overall survival (OS) at 2 years significantly improved over time. The improved OS among patients in ≥ complete remission (CR)3 and active disease at CBT was mainly due to a reduction of relapse-related mortality, whereas among patients in first or second CR at CBT, this was due mainly to a reduction of non-relapse mortality. The trends of neutrophil engraftment also improved over time. This experience demonstrated that the survival and engraftment rate after CBT for this group has improved over the past 22 years.

## Introduction

The availability of cryopreserved cord blood could allow patients without appropriate donors to receive allogeneic hematopoietic cell transplantation (HCT). This is because cord blood transplantation (CBT) is acceptable for tolerance to human leukocyte antigen (HLA) mismatch and is rapidly available [1–3]. Indeed, cord blood is an alternative donor source for adult acute myeloid leukemia (AML) patients without a matched related or unrelated donor [4–9]. In Japan, CBT from an unrelated donor has been performed in adults with AML since 1998 [10, 11]. The annual number of CBT exceeds 1300, and the cumulative number of CBT reached 18,242 in 2019, which accounted for more than 30% of the total CBT performed worldwide [3]. The majority of these cases were for the treatment of AML [11, 12].

The major limitations of CBT for adult patients are higher rates of engraftment failure and mortality, particularly in non-relapse mortality (NRM). Indeed, patients with advanced disease frequently received CBT, which might be associated with poor outcomes. Nevertheless, our recent study showed that early mortality and engraftment failure have improved after CBT as the first allogeneic HCT for 9678 adults over the past 20 years in Japan [11]. This was similar to the improvement of mortality after allogeneic HCT from adult donors for a relatively heterogeneous group of patients [13–18]. However, it might depend on disease status at HCT. Thus, large-scale data on CBT outcomes for unselected adult AML are lacking, but the data on CBT in Japan can be used to capture outcomes of CBT for adult AML patients in a real-world setting. Here, we analyzed the trends in survival and engraftment after single-unit CBT for unselected adult AML patients by using a nationwide Japanese database.

## Methods

### Data collection and study population

This retrospective study was conducted by the Adult AML Working Group of the Japanese Society for Transplantation and Cellular Therapy (JSTCT). The clinical data were collected by the Transplant Registry Unified Management Program (TRUMP) of the Japanese Data Center for Hematopoietic Cell Transplantation (JDCHCT) and the JSTCT [19, 20]. Patients aged ≥16 years who received unrelated single-unit CBT as first allogeneic HCT between 1998 and 2019 in Japan were eligible. Patients who received double-unit CBT (*n* = 95) or related CBT (*n* = 1) were excluded from this study. We also excluded patients that lacked data about survival status (*n* = 12), and those with a previous history of allogeneic HCT (*n* = 1670). Finally, 5504 patients were eligible for this study. The primary outcome was overall survival (OS). Secondary outcomes were relapse-related mortality, NRM, neutrophil and platelet engraftment, and acute and chronic graft-versus-host disease (GVHD). This study was approved by the adult AML working group of the JSTCT and by the institutional review board of the Institute of Medical Science, The University of Tokyo (2021-30-0729).

### Definition

OS was defined as death due to any cause measured from the date of CBT. Surviving patients were censored at 2 years after CBT. Relapse-related mortality was defined as death after a hematological recurrence of AML. NRM was defined as death without leukemia recurrence. The times of neutrophil or platelet engraftment were defined as the first of the three consecutive days when the absolute neutrophil count was higher than 0.5 × 10^9^/L or the platelet count was higher than 20 × 10^9^/L without platelet transfusion, respectively. The grading of acute and chronic GVHD was defined using standard criteria [21, 22]. Eastern Cooperative Oncology Group performance status (PS) [23], HCT-specific comorbidity index (HCT-CI) [24], and cytogenetic risk [25] were classified in accordance with published criteria. Complete remission (CR) was defined as less than 5% of bone marrow blasts and the absence of leukemic blasts in peripheral blood or extramedullary sites. Early phase at CBT was defined as CR1 or CR2. Advanced phase at CBT was defined as ≥CR3 and signs of active disease including primary induction failure, refractory relapse, and untreated disease. The intensity of conditioning was classified in accordance with published criteria [26]. The degree of HLA matching between donor and recipient was based on a low-resolution analysis for HLA-A, HLA-B, and HLA-DR.

### Statistical analysis

All statistical analyses were conducted with EZR (Saitama Medical Center, Jichi Medical University, Saitama, Japan), a graphical user interface for the R 4.1.2 software program (R Foundation for Statistical Computing, Vienna, Austria) [27]. Two-sided *P* values less than 0.05 were considered statistically significant.

Baseline characteristics between the three periods of CBT were compared using a chi-squared or Fisher exact test for categorical variables and the Kruskal–Wallis test for continuous variables.

The Kaplan–Meier method was used to estimate the probability of OS, which was compared using the log-rank test. Cumulative incidence estimates were used to calculate the competing risks outcomes, such as relapse-related mortality, NRM, neutrophil and platelet engraftment, and acute and chronic GVHD. These outcomes were compared using Gray’s test. Relapse-related mortality was a competing event for NRM and vice versa. For hematopoietic engraftment, death before hematopoietic engraftment was a competing event. For GVHD, death before the onset of GVHD was a competing risk. Multivariate analyses were performed using a Cox proportional hazards regression model for overall mortality (1-OS) and the Fine and Gray proportional hazards model for competing risk outcomes.

To adjust for differences in baseline characteristics, all possible confounding variables were considered for the multivariate analysis. These variables included age (<55 vs ≥55 years), HCT-CI (0–2 vs ≥3 vs unknown), cytogenetic risk (other than adverse vs adverse), prior history of myelodysplastic syndrome (MDS)/myeloproliferative neoplasm (MPN) (yes vs no), disease status at CBT (early phase vs. advance phase), cryopreserved cord blood total nucleated cell (TNC) count (<2.5 × 10^7^/kg vs ≥2.5 × 10^7^/kg), cryopreserved cord blood CD34^+^ cell count (<0.8 × 10^5^/kg vs ≥0.8 × 10^5^/kg), HLA disparities (≤1 vs ≥2 mismatch), ABO incompatibility (match, minor mismatch vs major, bidirectional mismatch), sex incompatibility (other than female donor to male recipient vs female donor to male recipient vs unknown), intensity of conditioning regimen (myeloablative conditioning [MAC] vs reduced-intensity conditioning [RIC]), use of total body irradiation [TBI] (non-TBI vs TBI), GVHD prophylaxis (methotrexate [MTX]-based vs. other than MTX-based), and time period of CBT (1998–2007 vs 2008–2013 vs 2014–2019). Missing data of HCT-CI and sex incompatibility were accounted for as separate categories. Cryopreserved cord blood TNC and CD34^+^ cell counts were divided according to an approximately median value. Results are expressed as the hazard ratio (HR) and 95% confidence interval (CI).

We evaluated the time periods of transplant outcomes in the entire cohort and separately evaluated them into two distinct patient cohorts based on disease status at CBT: (1) patients in CR1 and CR2 at CBT (early phase at CBT); and (2) patients in advanced disease status at CBT (advanced phase at CBT).

## Results

### Patient and transplant characteristics

A total of 5504 patients who received single-unit CBT for AML were included (Table 1). Among them, 1029, 1867, and 2608 patients were transplanted in 1998–2007, 2008–2013, and 2014–2019, respectively. Over the three time periods, there was a progressive increase in older age at CBT, adverse cytogenetics of AML, absence of MDS/MPN, early phase at CBT, cryopreserved TNC dose, cryopreserved CD34^+^ cell dose, MAC regimens, use of non-TBI regimens, and GVHD prophylaxis without MTX. The data for PS, HCT-CI, and anti-HLA antibody were mostly unavailable during the former time periods (1998–2007). Among 1096 patients who had anti-HLA antibody, 56 (5%) patients had donor-specific anti-HLA antibody (DSA).

**Table 1:** Patient and transplant characteristics.

	Entire cohort	1998–2007	2008–2013	2014–2019	*P*
Number of patients	5504	1029	1867	2608	
Age, median (IQR), years	54 (42–63)	49 (35–58)	55 (42–63)	56 (45–64)	<0.001
Age, number (%)					<0.001
16–54 years	2763 (50.2)	673 (65.4)	911 (48.8)	1179 (45.2)	
≥55 years	2741 (49.8)	356 (34.6)	956 (51.2)	1429 (54.8)	
Sex, number (%)					0.787
Male	3176 (57.7)	584 (56.8)	1081 (57.9)	1511 (58.0)	
Female	2327 (42.3)	445 (43.2)	786 (42.1)	1096 (42.0)	
Missing data	1	0	0	1	
Body weight*, median (IQR), kg	55.4 (48.9–62.8)	55.0 (48.5–61.7)	55.4 (48.8–62.4)	55.7 (49.0–63.4)	0.028
Performance status, number (%)					<0.001
0	4559 (82.8)	669 (65.0)	1564 (83.8)	2831 (89.2)	
≥2	699 (12.7)	123 (12.0)	583 (15.9)	486 (10.7)	
Missing data	246 (4.5)	237 (23.0)	7 (0.4)	2 (0.1)	
HCT-CI, number (%)					<0.001
0–2	3710 (67.4)	248 (24.1)	1459 (78.1)	2003 (76.8)	
≥3	999 (18.2)	53 (5.2)	365 (19.6)	581 (22.3)	
Missing data	795 (14.4)	728 (70.7)	43 (2.3)	24 (0.9)	
Recipient CMV status, number (%)					<0.001
Negative	866 (15.7)	123 (12.0)	368 (19.7)	375 (14.4)	
Positive	4266 (77.5)	757 (73.6)	1362 (73.0)	2147 (82.3)	
Missing data	372 (6.8)	149 (14.5)	137 (7.3)	86 (3.3)	
Anti HLA-antibody status, number (%)					<0.001
Negative	3133 (56.9)	237 (23.0)	1132 (60.6)	1764 (67.6)	
Positive	1096 (19.9)	23 (2.2)	327 (17.5)	746 (28.6)	
Donor-specific anti-HLA antibody (+)	56	1	17	38	
Donor-specific anti-HLA antibody (–)	1039	21	310	708	
Missing data	1	1	0	0	
Missing data	1275 (23.2)	769 (74.7)	408 (21.9)	98 (3.8)	
Cytogenetics, number (%)					<0.001
Other than adverse	4178 (75.9)	850 (82.6)	1407 (75.4)	1921 (73.7)	
Adverse	1326 (24.1)	179 (17.4)	460 (24.6)	687 (26.3)	
Prior history of MDS/MPN, number (%)					<0.001
Absence	4568 (83.0)	822 (79.9)	1516 (81.2)	2230 (85.5)	
Presence	936 (17.0)	207 (20.1)	351 (18.8)	378 (14.5)	
Disease status at CBT, number (%)					<0.001
CR1, CR2	2289 (42.5)	375 (37.6)	711 (38.8)	1203 (47.2)	
CR ≥ 3 relapse, induction failure, untreated	3091 (57.5)	622 (62.4)	1123 (61.2)	1346 (52.8)	
Missing data	124	32	33	59	
Cryopreserved TNC dose*, median (IQR), ×10^7^ cells/kg	2.61 (2.25–3.13)	2.44 (2.13–2.86)	2.62 (2.25–3.15)	2.67 (2.30–3.21)	<0.001
Cryopreserved TNC dose*, number (%)					<0.001
<2.5 × 10^7^ cells/kg	2299 (42.5)	530 (54.0)	763 (41.5)	1006 (38.8)	
≥2.5 × 10^7^ cells/kg	3113 (57.5)	452 (46.0)	1074 (58.5)	1587 (61.2)	
Missing data	92	47	30	15	
Cryopreserved CD34+ cells*, median (IQR), ×10^5^ cells/kg	0.83 (0.60–1.12)	0.76 (0.51–1.12)	0.83 (0.61–1.15)	0.84 (0.63–1.11)	<0.001
Cryopreserved CD34^+^ cells*, number (%)					<0.001
<0.8 × 10^5^ cells/kg	2548 (47.1)	517 (53.1)	846 (46.1)	1185 (45.6)	
≥0.8 × 10^5^ cells/kg	2859 (52.9)	457 (46.9)	991 (53.9)	1411 (54.4)	
Missing data	97	55	30	12	
HLA disparities, number (%)					0.640
0,1	2212 (40.5)	420 (41.7)	739 (39.9)	1053 (40.4)	
≥2	3249 (59.5)	586 (58.3)	1111 (60.1)	1552 (59.6)	
Missing data	43	23	17	3	
ABO incompatibility, number (%)					0.011
Match/Minor mismatch	3297 (65.2)	582 (58.4)	1174 (63.3)	1541 (59.4)	
Major/Bidirectional mismatch	2150 (39.5)	415 (41.6)	682 (36.7)	1053 (40.6)	
Missing data	57	32	11	14	
Sex incompatibility, number (%)					<0.001
Other than female donor to male recipient	3608 (65.6)	657 (63.8)	1122 (60.1)	1829 (70.1)	
Female donor to male recipient	1453 (26.4)	285 (27.7)	405 (21.7)	763 (29.3)	
Missing data	443 (8.0)	87 (8.5)	340 (18.2)	16 (0.6)	
Conditioning regimen, number (%)					<0.001
MAC	3580 (65.2)	578 (57.1)	1156 (62.0)	1846 (70.8)	
RIC	1907 (34.8)	435 (42.9)	710 (38.0)	762 (29.2)	
Missing data	17	16	1	0	
Use of TBI					<0.001
Non-TBI	1571 (28.6)	142 (13.9)	386 (20.7)	1043 (40.0)	
TBI	3920 (71.4)	877 (86.1)	1478 (79.3)	1565 (60.0)	
Missing data	13	10	3	0	
Use of ATG/ALG, number (%)					<0.001
ATG/ALG (–)	5326 (97.0)	1010 (99.3)	1802 (96.7)	2514 (96.4)	
ATG/ALG (+)	163 (3.0)	7 (0.7)	62 (3.3)	94 (3.6)	
Missing data	15	12	3	0	
GVHD prophylaxis, number (%)					<0.001
With MTX	3070 (56.2)	604 (60.0)	1082 (58.3)	1384 (53.2)	
Without MTX	2394 (43.8)	403 (40.0)	775 (41.7)	1216 (46.8)	
Missing data	40	22	10	8	

*IQR* interquartile range, *HCT-CI* hematopoietic cell transplantation-specific comorbidity index, *CMV* cytomegalovirus, *HLA* human leukocyte antigen, *MDS* myelodysplastic syndrome, *MPN* myeloproliferative neoplasm, *CBT* cord blood transplantation, *CR* complete remission, *HCT* hematopoietic cell transplantation, *TNC* total nucleated cell, *MAC* myeloablative conditioning, *RIC* reduced-intensity conditioning, *TBI* total body irradiation, *ATG* antithymocyte globulin, *ALG* antilymphocyte globulin, *GVHD* graft-versus-host disease, *MTX* methotrexate.

In the subset analysis, 2289, and 3091 patients with early phase at CBT, and advanced phase at CBT were analyzed, respectively (Table 2). The distributions of patients and transplantations in each cohort across the three time periods were similar to those of the entire cohort. For patients with early phase at CBT, RIC regimens were the most frequent during the middle time periods (2008–2013) (Table 2).

**Table 2:** Patient and transplant characteristics according to disease status at CBT.

	Early phase				Advanced phase			
	1998–2007	2008–2014	2015–2019	*P*	1998–2007	2008–2014	2015–2019	*P*
Number of patients	375	711	1203		622	1123	1346	
Age, median (IQR), years	45 (32–55)	53 (41–60)	54 (42–63)	<0.001	51 (37–60)	56 (44–64)	59 (48–65)	<0.001
Age, number (%)				<0.001				<0.001
16–54 years	274 (73.1)	376 (52.9)	610 (50.7)		378 (60.8)	515 (45.9)	529 (39.3)	
≥55 years	101 (26.9)	335 (47.1)	593 (49.3)		244 (39.2)	608 (54.1)	817 (60.7)	
Sex, number (%)				0.456				0.783
Male	193 (51.5)	394 (55.4)	654 (54.4)		373 (60.0)	669 (59.6)	820 (60.9)	
Female	182 (48.5)	317 (44.6)	548 (45.6)		249 (40.0)	454 (40.4)	526 (39.1)	
Missing data	0	0	1		0	0	0	
Body weight*, median (IQR), kg	55.0 (48.0–61.6)	56.0 (49.0–62.6)	56.0 (48.9–63.6)	0.097	55.0 (49.0–61.7)	55.0 (48.7–62.1)	55.4 (49.0–63.4)	0.181
Performance status, number (%)				<0.001				<0.001
0	285 (76.0)	682 (95.9)	1152 (95.8)		373 (60.0)	856 (76.2)	1118 (83.1)	
≥2	14 (3.7)	28 (3.9)	51 (4.2)		106 (17.0)	262 (23.3)	226 (16.8)	
Missing data	76 (20.3)	1 (0.1)	0		143 (23.0)	5 (0.4)	2 (0.1)	
HCT-CI, number (%)				<0.001				<0.001
0–2	85 (22.7)	610 (85.8)	985 (81.9)		159 (25.6)	821 (73.1)	964 (71.6)	
≥3	14 (3.7)	90 (12.7)	212 (17.6)		37 (5.9)	272 (24.2)	364 (27.0)	
Missing data	276 (73.6)	11 (1.5)	6 (0.5)		426 (68.5)	30 (2.7)	18 (1.3)	
Recipient CMV status, number (%)				<0.001				<0.001
Negative	53 (14.1)	137 (19.3)	189 (15.7)		66 (10.6)	224 (19.9)	178 (13.2)	
Positive	279 (74.4)	517 (72.7)	981 (81.5)		462 (74.3)	823 (73.3)	1115 (82.8)	
Missing data	43 (11.5)	57 (8.0)	33 (2.7)		94 (15.1)	76 (6.8)	53 (3.9)	
Anti HLA-antibody status, number (%)				<0.001				<0.001
Negative	90 (24.0)	451 (63.4)	849 (70.6)		143 (23.0)	658 (58.6)	869 (64.6)	
Positive	11 (2.9)	96 (13.5)	314 (26.1)		12 (1.9)	229 (20.4)	420 (31.2)	
Missing data	274 (73.1)	164 (23.1)	40 (3.3)		467 (75.1)	236 (21.0)	57 (4.2)	
Cytogenetics, number (%)				0.049				<0.001
Other than adverse	325 (86.7)	586 (82.4)	976 (81.1)		493 (79.3)	788 (70.2)	886 (65.8)	
Adverse	50 (13.3)	125 (17.6)	227 (18.9)		129 (20.7)	335 (29.8)	460 (34.2)	
Prior history of MDS/MPN, number (%)				0.119				0.019
Absence	335 (89.3)	644 (90.6)	1112 (92.4)		460 (74.0)	839 (74.7)	1060 (78.8)	
Presence	40 (10.7)	67 (9.4)	91 (7.6)		162 (26.0)	284 (25.3)	286 (21.2)	
Cryopreserved TNC dose*, median (IQR), ×10^7^ cells/kg	2.45 (2.13–2.83)	2.60 (2.24–3.09)	2.68 (2.31–3.20)	<0.001	2.44 (2.13–2.86)	2.62 (2.26–3.17)	2.66 (2.29–3.23)	<0.001
Cryopreserved TNC dose*, number (%)				<0.001				<0.001
<2.5 × 10^7^ cells/kg	192 (53.3)	296 (42.6)	460 (38.5)		327 (54.3)	461 (41.5)	525 (39.2)	
≥2.5 × 10^7^ cells/kg	168 (46.7)	399 (57.4)	735 (61.5)		275 (45.7)	650 (58.5)	814 (60.8)	
Missing data	15	16	8		20	12	7	
Cryopreserved CD34+ cells*, median (IQR), ×10^5^ cells/kg	0.73 (0.49–1.15)	0.83 (0.61–1.18)	0.85 (0.63–1.13)	0.002	0.77 (0.51–1.10)	0.83 (0.61–1.14)	0.83 (0.62–1.09)	0.003
Cryopreserved CD34^+^ cells*, number (%)				0.004				0.034
<0.8 × 10^5^ cells/kg	195 (54.5)	315 (45.4)	536 (44.8)		314 (52.5)	517 (46.5)	625 (46.6)	
≥0.8 × 10^5^ cells/kg	163 (45.5)	379 (54.6)	660 (55.2)		284 (47.5)	594 (53.5)	716 (53.4)	
Missing data	17	17	7		24	12	5	
HLA disparities, number (%)				0.759				0.165
0,1	146 (39.2)	291 (41.4)	496 (41.3)		265 (43.3)	432 (38.7)	532 (39.6)	
≥2	226 (60.8)	412 (58.6)	706 (58.7)		347 (56.7)	683 (61.3)	812 (60.4)	
Missing data	3	8	1		10	8	2	
ABO incompatibility, number (%)				0.098				0.031
Match/ Minor mismatch	231 (62.1)	455 (64.4)	713 (59.5)		337 (55.8)	696 (62.3)	792 (59.2)	
Major/ Bidirectional mismatch	141 (37.9)	251 (35.6)	485 (40.5)		267 (44.2)	422 (37.7)	545 (40.8)	
Missing data	3	5	5		18	5	9	
Sex incompatibility, number (%)				<0.001				<0.001
Other than female donor to male recipient	255 (68.0)	431 (60.6)	862 (71.7)		384 (61.7)	669 (59.6)	922 (68.5)	
Female donor to male recipient	95 (25.3)	150 (21.1)	336 (27.9)		185 (29.7)	252 (22.4)	413 (30.7)	
Missing data	25 (6.7)	130 (18.3)	5 (0.4)		53 (8.5)	202 (18.0)	11 (0.8)	
Conditioning regimen, number (%)				<0.001				<0.001
MAC	248 (66.1)	423 (59.5)	821 (68.2)		313 (51.0)	723 (64.4)	985 (73.2)	
RIC	127 (33.9)	288 (40.5)	382 (31.8)		301 (49.0)	399 (35.6)	361 (26.8)	
Missing data	0	0	0		8	1	0	
Use of TBI				<0.001				<0.001
Non-TBI	44 (11.7)	91 (12.8)	361 (30.0)		96 (15.5)	288 (25.7)	671 (49.9)	
TBI	331 (88.3)	618 (87.2)	842 (70.0)		523 (84.5)	834 (74.3)	675 (50.1)	
Missing data	0	2	0		3	1	0	
Use of ATG/ALG, number (%)				0.005				0.001
ATG/ALG (–)	373 (99.5)	688 (97.0)	1157 (96.2)		614 (99.4)	1081 (96.3)	1299 (96.5)	
ATG/ALG (+)	2 (0.5)	21 (3.0)	46 (3.8)		4 (0.6)	41 (3.7)	47 (3.5)	
Missing data	0	2	0		4	1	0	
GVHD prophylaxis, number (%)				<0.001				<0.001
With MTX	266 (71.5)	479 (67.8)	737 (61.4)		327 (53.3)	580 (51.8)	605 (45.1)	
Without MTX	106 (28.5)	227 (32.2)	464 (38.6)		286 (46.7)	539 (48.2)	736 (54.9)	
Missing data	3	5	2		9	4	5	

*IQR* interquartile range, *HCT-CI* hematopoietic cell transplantation-specific comorbidity index, *CMV* cytomegalovirus, *HLA* human leukocyte antigen, *MDS* myelodysplastic syndrome, *MPN* myeloproliferative neoplasm, *CBT* cord blood transplantation, *CR* complete remission, *TNC* total nucleated cell, *MAC* myeloablative conditioning, *RIC* reduced-intensity conditioning, *TBI* total body irradiation, *ATG* antithymocyte globulin, *ALG* antilymphocyte globulin, *GVHD* graft-versus-host disease, *MTX* methotrexate.

### OS

**Fig. 1:**
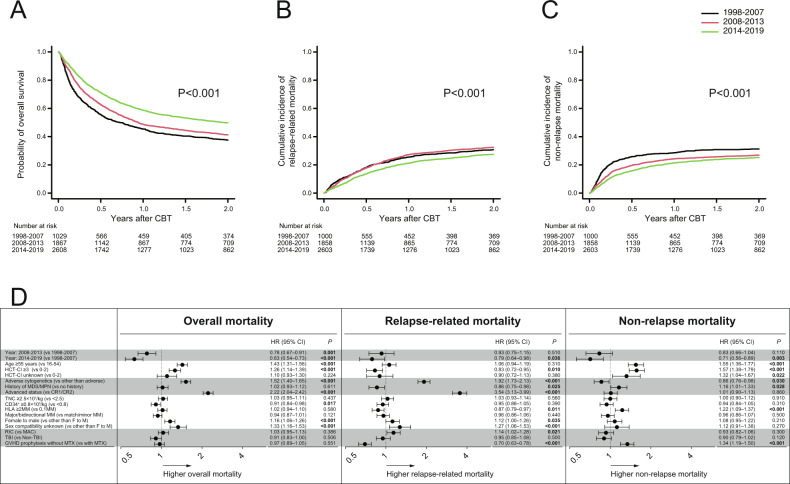
Overall survival, relapse-related moratlity, and non-relapse mortality after CBT in the entire cohort. The probability of overall survival (**A**) and the cumulative incidences of relapse-related mortality (**B**) and non-relapse mortality (**C**) after CBT according to the three time periods in the entire cohort. Forest plots of the adjusted hazard ratios (HR) and 95% confidence intervals (CI) of overall mortality, relapse-related mortality, and non-relapse mortality in the multivariate analysis (**D**).

Among the entire cohort, the probability of 2-year OS was 44.5% (95%CI, 43.1–45.9%). In the univariate analysis, the 2-year OS significantly improved over time (37.5% for 1998–2007, 41.2% for 2008–2013, and 49.8% for 2014–2019, *P* < 0.001 by log-rank test) (Fig. 1A). In the multivariate analysis, compared with 1998–2007, overall mortality was significantly lower during the periods 2008–2013 (HR,0.78, *P* = 0.001) and 2014–2019 (HR,0.63, *P* < 0.001) (Fig. 1D). In relation to other factors associated with overall mortality, age ≥55 years (HR,1.43, *P* < 0.001), HCT-CI ≥ 3 (HR,1.26, *P* < 0.001), adverse cytogenetic risk (HR,1.52, *P* < 0.001), advanced phase at CBT (HR,2.22, *P* < 0.001), cryopreserved cord blood CD34^+^ cell count ≥0.8 × 10^5^/kg (HR,0.91, *P* = 0.017), female donor to male recipient (HR,1.16, *P* < 0.001), and unknown status of sex incompatibility (HR,1.33, P < 0.001) were also significantly associated with overall mortality (Fig. 1D).

**Fig. 2:**
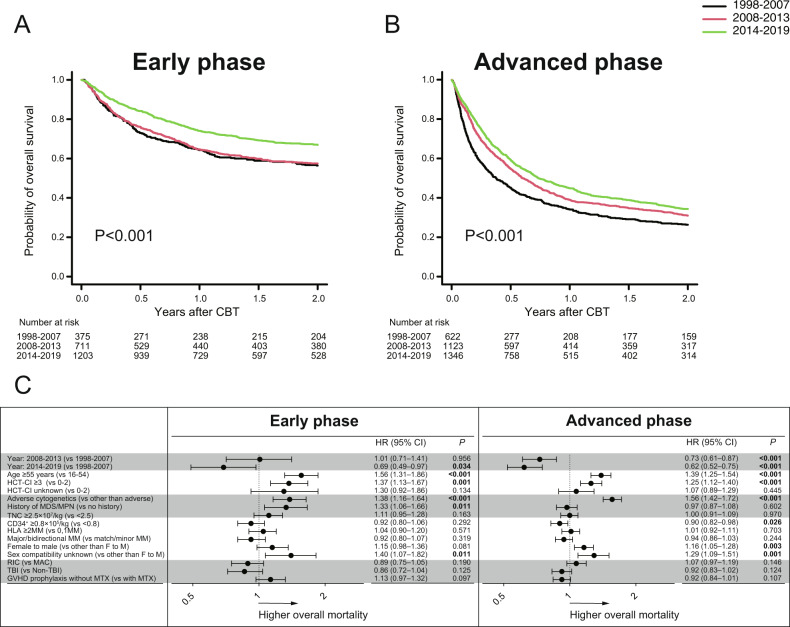
Overall survival after CBT according to disease status at CBT. The probabilities of overall survival after CBT according to the three time periods in patients with early phase at CBT (**A**), or advanced phase at CBT (**B**). Forest plots of the adjusted hazard ratios (HR) and 95% confidence intervals (CI) of overall mortality among each cohort in the multivariate analysis (**C**).

Among the distinct cohorts, in the univariate analysis, the 2-year OS significantly improved over time (*P* < 0.001 for early phase at CBT; and *P* < 0.001 for advanced phase at CBT) (Fig. 2A, B). In the multivariate analysis, compared with 1998–2007, overall mortality was significantly lower during the recent time period 2014–2019 (HR,0.69, *P* = 0.034 for early phase at CBT; HR,0.62, *P* < 0.001 for advanced phase at CBT) in two cohorts (Fig. 2C). However, overall mortality was significantly lower during the middle time period 2008–2013 only among patients with advanced phase at CBT (HR,0.73, *P* < 0.001) but not with early phase at CBT (HR,1.01, *P* = 0.956) (Fig. 2C).

### Relapse-related mortality

Among the entire population, the probability of 2-year relapse-related mortality was 29.8% (95%CI, 28.6–31.1%). The 2-year relapse-related mortality was 30.8% for 1998–2007, 32.5% for 2008–2013, and 27.5% for 2014–2019 (*P* < 0.001 by Gray’s test) (Fig. 1B). In the multivariate analysis, compared with 1998–2007, relapse-related mortality was significantly lower in the last period 2014–2019 (HR,0.79, *P* = 0.036) but not 2008–2013 (HR,0.93, *P* = 0.510) (Fig. 1D). In relation to other factors associated with relapse-related mortality, adverse cytogenetic risk (HR,1.92, *P* < 0.001), history of MDS/MPN (HR,0.86, *P* = 0.025), advanced phase at CBT (HR,3.54, *P* < 0.001), HLA disparities ≥2 mismatch (HR,0.87, *P* = 0.011), female donor to male recipient (HR,1.12, *P* = 0.035), unknown status of sex incompatibility (HR,1.27, *P* < 0.001), RIC regimens (HR,1.14, *P* = 0.021), and GVHD prophylaxis without MTX (HR,0.70, *P* < 0.001) were also significantly associated with relapse-related mortality (Fig. 1D).

**Fig. 3:**
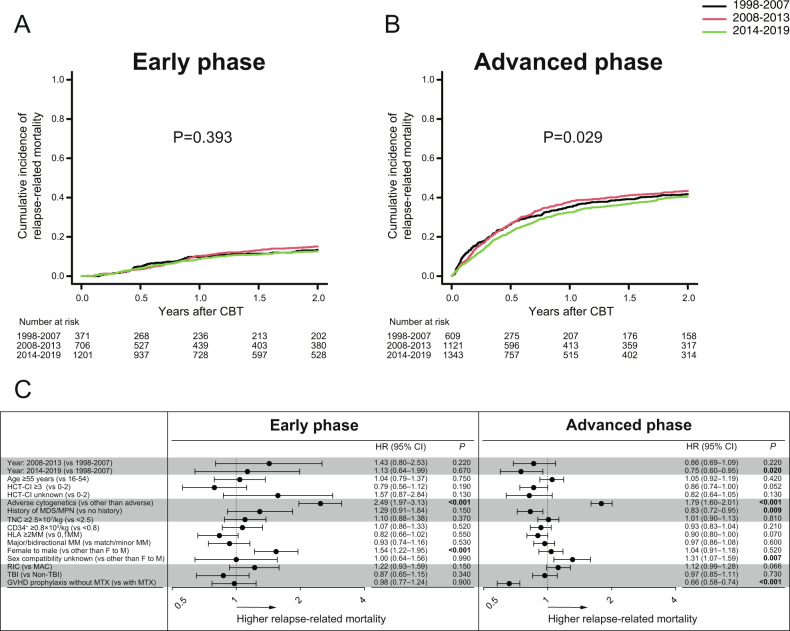
Relapse-related mortality after CBT according to disease status at CBT. The cumulative incidences of relapse-related mortality after CBT according to the three time periods in patients with early phase at CBT (**A**), or advanced phase at CBT (**B**). Forest plots for the adjusted hazard ratios (HR) and 95% confidence intervals (CI) of relapse-related mortality among each cohort in the multivariate analysis (**C**).

Among two distinct cohorts, in the univariate analysis, the 2-year relapse-related mortality significantly improved over time among patients with advanced phase at CBT (*P* = 0.029), but not those with early phase at CBT (*P* = 0.393) (Fig. 3A, B). In the multivariate analysis, compared with 1998–2007, relapse-related mortality was significantly lower in the recent period 2014–2019 among patients with advanced phase at CBT (HR,0.75, *P* = 0.020) (Fig. 3C).

### NRM

The probability of 2-year NRM was 26.8% (95%CI, 25.6–28.0%) for the entire cohort. The 2-year NRM was 31.3% for 1998–2007, 26.9% for 2008–2013, and 25.2% for 2014–2019 (*P* < 0.001) (Fig. 1C). In the multivariate analysis, compared with 1998–2007, NRM was significantly lower in the last period 2014–2019 (HR,0.71, *P* = 0.003) but not 2008–2013 (HR,0.83, *P* = 0.110) (Fig. 1D). In relation to other factors associated with NRM, age ≥55 years (HR,1.55, *P* < 0.001), HCT-CI ≥ 3 (HR,1.57, *P* < 0.001), unknown status of HCT-CI (HR,1.32, *P* = 0.022), adverse cytogenetic risk (HR,0.86, *P* = 0.030), history of MDS/MPN (HR,1.16, *P* = 0.028), HLA disparities ≥2 mismatch (HR,1.22, *P* < 0.001), and GVHD prophylaxis without MTX (HR,1.34, *P* < 0.001) were also significantly associated with NRM (Fig. 1D).

**Fig. 4:**
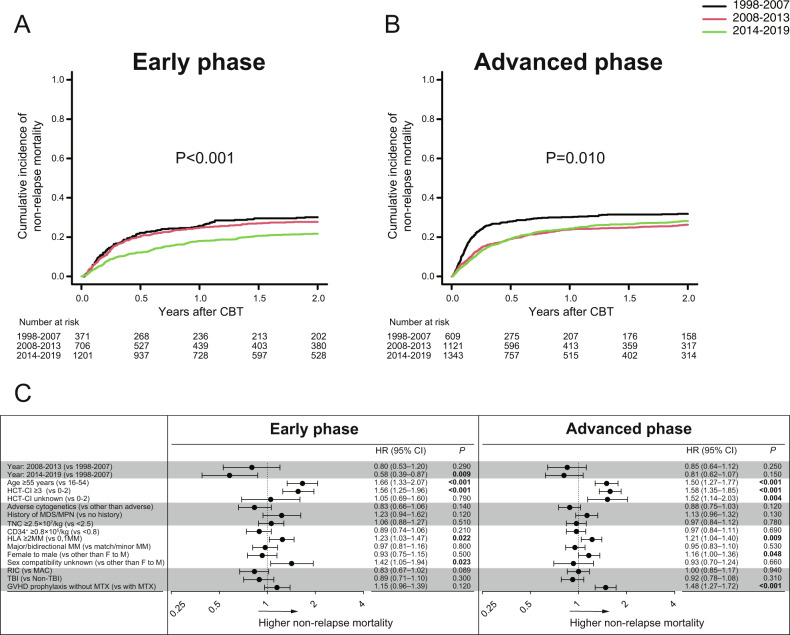
Non-relapse mortality after CBT according to disease status at CBT. The cumulative incidences of non-relapse mortality after CBT according to the three time periods in patients with early phase at CBT (**A**), or advanced phase at CBT (**B**). Forest plots of the adjusted hazard ratios (HR) and 95% confidence intervals (CI) of non-relapse mortality among each cohort in the multivariate analysis (**C**).

Among two distinct cohorts, in the univariate analysis, the 2-year NRM significantly improved over time among patients with early phase at CBT (*P* < 0.001) and advanced phase at CBT (*P* = 0.010) (Fig. 4A, B). In the multivariate analysis, compared with 1998–2007, NRM was significantly lower in the last period 2014–2019 among patients with early phase at CBT (HR,0.58, *P* = 0.009) (Fig. 4C).

### Hematopoietic engraftments

For the entire cohort, the cumulative incidences of neutrophil and platelet engraftment were 80.0% (95%CI, 78.9–81.0%) at 42 days and 65.1% (95%CI, 63.8–66.3%) at 100 days after CBT, respectively. Improvements of neutrophil and platelet engraftment were observed in the periods 2008–2013 and 2014–2019 compared with 1998–2007 in the univariate and multivariate analyses (Fig. S1A–C).

**Fig. 5:**
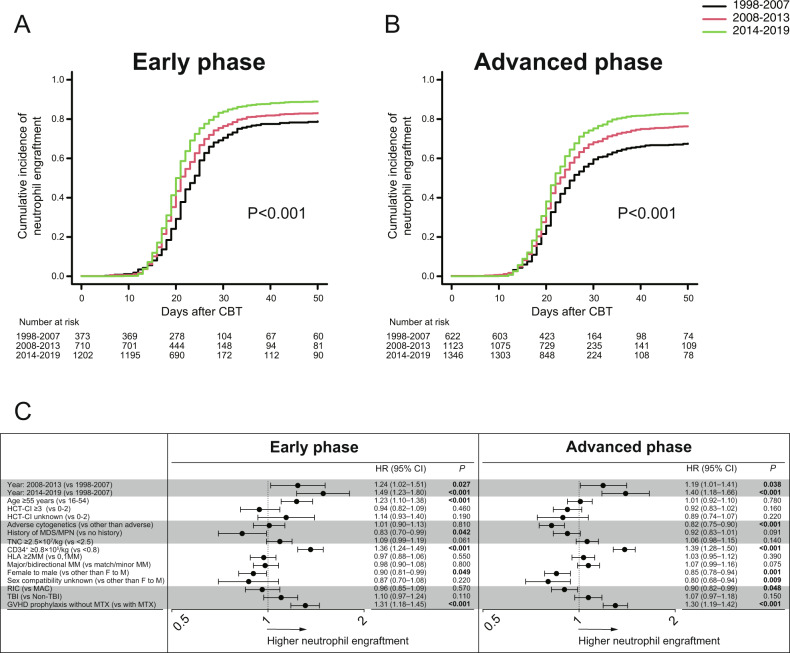
Neutrophil engraftment after CBT according to disease status at CBT. The cumulative incidences of neutrophil engraftment after CBT according to the three time periods in patients with early phase at CBT (**A**), or advanced phase at CBT (**B**). Forest plots of the adjusted hazard ratios (HR) and 95% confidence intervals (CI) of neutrophil engraftment among each cohort in the multivariate analysis (**C**).

Among two distinct cohorts, in the univariate analysis, neutrophil engraftment significantly improved over time (*P* < 0.001 for early phase at CBT; *P* < 0.001; and for advanced phase at CBT) (Fig. 5A, B). In the multivariate analysis, compared with 1998–2007, neutrophil engraftment was significantly higher in the last period 2014–2019 (HR,1.49, *P* < 0.001 for early phase at CBT; HR,1.40, *P* < 0.001 for advanced phase at CBT) in all three cohorts (Fig. 5C), and in the period 2008–2013 among patients with early phase at CBT (HR,1.24, *P* = 0.027) and those with advanced phase at CBT (HR,1.19, *P* = 0.038) (Fig. 5C).

### GVHD

Among the entire cohort, the cumulative incidences of grades II–IV and III–IV acute GVHD were 35.6% (95% CI, 34.3–36.8%) and 11.4% (95% CI, 10.6–12.3%) at 100 days after CBT, respectively (Fig. S2A, S2B). Two years after CBT, the cumulative incidences of chronic and extensive chronic GVHD were 21.7% (95% CI, 20.6–22.8%) and 9.1% (95% CI, 8.4–9.9%), respectively (Fig. S3A, S3B). Except for the cumulative incidence of extensive chronic GVHD in the univariate analysis, which decreased over time (*P* = 0.003), the univariate and multivariate analyses showed that acute and chronic GVHD was not significantly different across the time periods (Fig. S2C,S3C).

Among two distinct cohorts, the multivariate analysis showed that acute and chronic GVHD was not significantly different across the time periods, except that grade III–IV acute GVHD was significantly lower in the middle time period (2008–2013) compared with 1998–2007 only among patients with early phase at CBT (HR,0.57, *P* = 0.047) (data not shown).

## Discussion

Our registry-based study assessed the trends in survival and engraftment after CBT for adult AML in a real-world setting. OS in the entire cohort significantly improved over time. Improved OS among patients with advanced phase at CBT was mainly due to the reduction of relapse-related mortality, whereas patients with early phase at CBT was mainly due to the reduction of NRM. Interestingly, trends of neutrophil engraftment were also improved over time for the two cohorts.

It can be speculated that an initial poor prognosis after CBT for adults might be partially attributed to a higher proportion of high-risk patients, including advanced disease status at CBT and adverse cytogenetics. Indeed, the proportion of advanced disease status at CBT was higher in both the former (1998-2007) and the middle (2008–2013) time periods in our cohort. However, improvements in survival were observed in each patient cohort based on disease status. This suggests that an initial poor prognosis of CBT for adults is independent of advanced disease status at CBT. Furthermore, although the proportion of patients with adverse cytogenetics increased over time throughout all cohorts, relapse-related mortality was significantly improved in the recent time period in patients with advanced phase at CBT but was not improved in patients with early phase at CBT. Although a reduction in the risk of relapse could depend on both the intensity of conditioning regimen [28, 29] and the strength of the graft-versus-leukemia (GVL) effect [30, 31], an improvement of relapse-related mortality over time is not entirely clear in patients with advanced phase at CBT. This is likely due to a significantly increased MAC regimen rather than enhanced a GVL effect because there was a progressive increase in MAC regimens over the three time periods among patients with advanced phase at CBT, but the incidences of acute and chronic GVHD did not change. Indeed, recent studies demonstrated that a higher intensity of conditioning regimen is preferred for CBT [28, 29, 32, 33]. Therefore, the strength of conditioning intensity could contribute to the improvement of relapse-related mortality.

Our previous study demonstrated a significant improvement of early NRM after CBT as the first allogeneic HCT for adults aged between 16 and 70 years with various hematological diseases over the past 20 years [11]. Our current study focusing adult AML also showed a significant decrease in NRM but only among patients with early phase at CBT in multivariate analysis. The reasons for this improvement of NRM are not clearly defined, but several factors, such as advances in supportive care, less toxic conditioning regimens, and more careful cord blood graft selection, along with higher achievements of neutrophil engraftment, could have contributed to the improvement of NRM. Importantly, the improvement of NRM was observed in the recent time period (2014–2019) but only among patients with remission status at CBT. This indicates that the improved NRM can be attributed mainly to the prevention, diagnosis, and management of infection, organ toxicity, and GVHD after CBT.

The most important limitation of CBT for adults is that the frequency of primary graft failure is higher after CBT compared with allogeneic HCT from adult donors [4]. Although the achievements of neutrophil engraftment after CBT could not completely attain the levels of those after allogeneic HCT from adult donors, trends of neutrophil engraftment were improved over time throughout all cohorts. The reasons for these trends in neutrophil engraftment are not entirely clear but could be attributed to the recent progress of a higher TNC and CD34^+^ cell dose in the cryopreserved cord blood unit [11]. This is partly due to improvements of collection and processing techniques of cryopreserved cord blood by cell processing technicians and obstetricians. Moreover, the presence of anti-HLA antibodies, particularly DSA, has mostly been evaluated before selection of a cord blood unit in recent time periods to avoid engraftment failure [8, 34]. Indeed, only 56 patients had DSA in the entire our cohort, which was too small population to clarify the impact of presence of DSA on engraftment failure. However, the data for anti-HLA antibodies were often unavailable in the former time period (1998–2007), suggesting that there might be a hidden existence of DSA. All these findings may account for some of the recent improvements of neutrophil engraftment over time for the all cohorts.

Our study had several limitations. First, data for the mutation profile of AML were insufficient in our registry data, which could strongly affect the outcomes after CBT for AML [35]. Second, we were unable to evaluate the advanced practices in the prevention and treatment of infection, particularly fungal and viral infections. Several studies demonstrated that new antifungal therapies, which have been approved for prophylaxis and treatment during the study period in Japan, could contribute to the improvement of survival after allogeneic HCT [36, 37]. Third, previous registry-based studies showed that high-volume center experience, which was defined as 20 or more annual numbers of unrelated CBT, was associated with better survival after CBT [38, 39]. However, the data for a center effect were unavailable in our registry data. Despite such limitations, the strength of this study was the largest number of unselected adult patients with AML undergoing single CBT, which could provide real-world data to clarify the efficacy and safety of CBT for this group.

In summary, our registry-based study under real-world settings demonstrated that the survival and engraftment rate after CBT for adult patients with AML has improved over the past 22 years. The causes are likely to be multifactorial including the recent progress of cord blood unit selection, conditioning regimen, and improvements of supportive care. However, mortality after CBT still has room for further improvement. Therefore, our real-world experience can support the next major approaches to reduce mortality after CBT for adult patients with AML.

## Supplementary information

Supplementary Figure 1

Supplementary Figure 2

Supplementary Figure 3

## Data Availability

Raw data were created by the TRUMP of the JDCHCT. The data that support the findings of this study are available from the corresponding author upon reasonable request.

## References

[1] Rocha V, Gluckman E, Eurocord-Netcord registry and European Blood and Marrow Transplant group. (2009). Improving outcomes of cord blood transplantation: HLA matching, cell dose and other graft- and transplantation-related factors. Br J Haematol.

[2] Ballen KK, Gluckman E, Broxmeyer HE (2013). Umbilical cord blood transplantation: the first 25 years and beyond. Blood..

[3] Yamamoto H (2019). Single cord blood transplantation in Japan; expanding the possibilities of CBT. Int J Hematol.

[4] Eapen M, Rocha V, Sanz G, Scaradavou A, Zhang MJ, Arcese W (2010). Effect of graft source on unrelated donor haemopoietic stem-cell transplantation in adults with acute leukaemia: a retrospective analysis. Lancet Oncol.

[5] Milano F, Gooley T, Wood B, Woolfrey A, Flowers ME, Doney K (2016). Cord-Blood Transplantation in Patients with Minimal Residual Disease. N. Engl J Med.

[6] Shouval R, Fein JA, Labopin M, Kröger N, Duarte RF, Bader P (2019). Outcomes of allogeneic haematopoietic stem cell transplantation from HLA-matched and alternative donors: a European Society for Blood and Marrow Transplantation registry retrospective analysis. Lancet Haematol.

[7] Miyao K, Terakura S, Kimura F, Konuma T, Miyamura K, Yanada M (2020). Updated comparison of 7/8 HLA allele-matched unrelated bone marrow transplantation and single-unit umbilical cord blood transplantation as alternative donors in adults with acute leukemia. Biol Blood Marrow Transpl.

[8] Konuma T, Kanda J, Yamasaki S, Harada K, Shimomura Y, Terakura S (2021). Single cord blood transplantation versus unmanipulated haploidentical transplantation for adults with acute myeloid leukemia in complete remission. Transpl Cell Ther.

[9] Shimomura Y, Sobue T, Hirabayashi S, Kondo T, Mizuno S, Kanda J (2022). Comparing cord blood transplantation and matched related donor transplantation in non-remission acute myeloid leukemia. Leukemia..

[10] Nagayama H, Nakayama K, Yasuo K, Tajika K, Dan K, Yamashita N (1999). Immunological reconstitution after cord blood transplantation for an adult patient. Bone Marrow Transpl.

[11] Konuma T, Kanda J, Inamoto Y, Hayashi H, Kobayashi S, Uchida N (2020). Improvement of early mortality in single-unit cord blood transplantation for Japanese adults from 1998 to 2017. Am J Hematol.

[12] Yanada M, Konuma T, Kuwatsuka Y, Kondo T, Kawata T, Takahashi S (2019). Unit selection for umbilical cord blood transplantation for adults with acute myeloid leukemia in complete remission: a Japanese experience. Bone Marrow Transpl.

[13] Gooley TA, Chien JW, Pergam SA, Hingorani S, Sorror ML, Boeckh M (2010). Reduced mortality after allogeneic hematopoietic-cell transplantation. N Engl J Med.

[14] Horan JT, Logan BR, Agovi-Johnson MA, Lazarus HM, Bacigalupo AA, Ballen KK (2011). Reducing the risk for transplantation-related mortality after allogeneic hematopoietic cell transplantation: how much progress has been made?. J Clin Oncol.

[15] Hahn T, McCarthy PL, Hassebroek A, Bredeson C, Gajewski JL, Hale GA (2013). Significant improvement in survival after allogeneic hematopoietic cell transplantation during a period of significantly increased use, older recipient age, and use of unrelated donors. J Clin Oncol.

[16] Majhail NS, Chitphakdithai P, Logan B, King R, Devine S, Rossmann SN (2015). Significant improvement in survival after unrelated donor hematopoietic cell transplantation in the recent era. Biol Blood Marrow Transpl.

[17] Canaani J, Beohou E, Labopin M, Ghavamzadeh A, Beelen D, Hamladji RM (2019). Trends in patient outcome over the past two decades following allogeneic stem cell transplantation for acute myeloid leukaemia: an ALWP/EBMT analysis. J Intern Med.

[18] Yanada M, Masuko M, Mori J, Aoki J, Mizuno S, Fukuda T (2019). Patients with acute myeloid leukemia undergoing allogeneic hematopoietic cell transplantation: trends in survival during the past two decades. Bone Marrow Transpl.

[19] Atsuta Y (2016). Introduction of Transplant Registry Unified Management Program 2 (TRUMP2): scripts for TRUMP data analyses, part I (variables other than HLA-related data). Int J Hematol.

[20] Kanda J (2016). Scripts for TRUMP data analyses. Part II (HLA-related data): statistical analyses specific for hematopoietic stem cell transplantation. Int J Hematol.

[21] Przepiorka D, Weisdorf D, Martin P, Klingemann HG, Beatty P, Hows J (1995). 1994 Consensus Conference on Acute GVHD Grading. Bone Marrow Transpl.

[22] Sullivan KM, Agura E, Anasetti C, Appelbaum F, Badger C, Bearman S (1991). Chronic graft-versus-host disease and other late complications of bone marrow transplantation. Semin Hematol.

[23] Oken MM, Creech RH, Tormey DC, Horton J, Davis TE, McFadden ET (1982). Toxicity and response criteria of the Eastern Cooperative Oncology Group. Am J Clin Oncol.

[24] Sorror ML, Maris MB, Storb R, Baron F, Sandmaier BM, Maloney DG (2005). Hematopoietic cell transplantation (HCT)-specific comorbidity index: a new tool for risk assessment before allogeneic HCT. Blood..

[25] Yanada M, Mori J, Aoki J, Harada K, Mizuno S, Uchida N (2018). Effect of cytogenetic risk status on outcomes for patients with acute myeloid leukemia undergoing various types of allogeneic hematopoietic cell transplantation: an analysis of 7812 patients. Leuk Lymphoma.

[26] Giralt S, Ballen K, Rizzo D, Bacigalupo A, Horowitz M, Pasquini M (2009). Reduced-intensity conditioning regimen workshop: defining the dose spectrum. Report of a workshop convened by the center for international blood and marrow transplant research. Biol Blood Marrow Transpl.

[27] Kanda Y (2013). Investigation of the freely available easy-to-use software ‘EZR’ for medical statistics. Bone Marrow Transpl.

[28] Konuma T, Ooi J, Uchida N, Ogawa H, Ohashi K, Kanamori H (2014). Granulocyte colony-stimulating factor combined regimen in cord blood transplantation for acute myeloid leukemia: a nationwide retrospective analysis in Japan. Haematologica..

[29] Arai Y, Takeda J, Aoki K, Kondo T, Takahashi S, Onishi Y (2015). Efficiency of high-dose cytarabine added to CY/TBI in cord blood transplantation for myeloid malignancy. Blood..

[30] Kanda J, Morishima Y, Terakura S, Wake A, Uchida N, Takahashi S (2017). Impact of graft-versus-host disease on outcomes after unrelated cord blood transplantation. Leukemia..

[31] Konuma T, Kanda J, Kuwatsuka Y, Yanada M, Kondo T, Hirabayashi S (2021). Differential effect of graft-versus-host disease on survival in acute leukemia according to donor type. Clin Cancer Res.

[32] Barker JN, Kurtzberg J, Ballen K, Boo M, Brunstein C, Cutler C (2017). Optimal practices in unrelated donor cord blood transplantation for hematologic malignancies. Biol Blood Marrow Transpl.

[33] Sharma P, Pollyea DA, Smith CA, Purev E, Kamdar M, Haverkos B (2018). Thiotepa-based intensified reduced-intensity conditioning adult double-unit cord blood hematopoietic stem cell transplantation results in decreased relapse rate and improved survival compared with transplantation following standard reduced-intensity conditioning: a retrospective cohort comparison. Biol Blood Marrow Transpl.

[34] Fuji S, Oshima K, Ohashi K, Sawa M, Saito T, Eto T (2020). Impact of pretransplant donor-specific anti-HLA antibodies on cord blood transplantation on behalf of the Transplant Complications Working Group of Japan Society for Hematopoietic Cell Transplantation. Bone Marrow Transpl.

[35] Papaemmanuil E, Gerstung M, Bullinger L, Gaidzik VI, Paschka P, Roberts ND (2016). Genomic classification and prognosis in acute myeloid leukemia. N Engl J Med.

[36] Spees LP, Martin PL, Kurtzberg J, Stokhuyzen A, McGill L, Prasad VK (2019). Reduction in mortality after umbilical cord blood transplantation in children over a 20-year period (1995-2014). Biol Blood Marrow Transpl.

[37] McDonald GB, Sandmaier BM, Mielcarek M, Sorror M, Pergam SA, Cheng GS (2020). Survival, nonrelapse mortality, and relapse-related mortality after allogeneic hematopoietic cell transplantation: comparing 2003-2007 versus 2013-2017 cohorts. Ann Intern Med..

[38] Shouval R, Ruggeri A, Labopin M, Mohty M, Sanz G, Michel G (2017). An integrative scoring system for survival prediction following umbilical cord blood transplantation in acute leukemia. Clin Cancer Res.

[39] Kanda J, Hayashi H, Ruggeri A, Kimura F, Volt F, Takahashi S (2020). Prognostic factors for adult single cord blood transplantation among European and Japanese populations: the Eurocord/ALWP-EBMT and JSHCT/JDCHCT collaborative study. Leukemia..

