# Supplementary figures and images for: Improved trends in survival and engraftment after single cord blood transplantation for adult acute myeloid leukemia
Source: Blood Cancer J. 2022 May 25;12(5):81. doi: 10.1038/s41408-022-00678-6 (PMC9132934; doi:10.1038/s41408-022-00678-6)

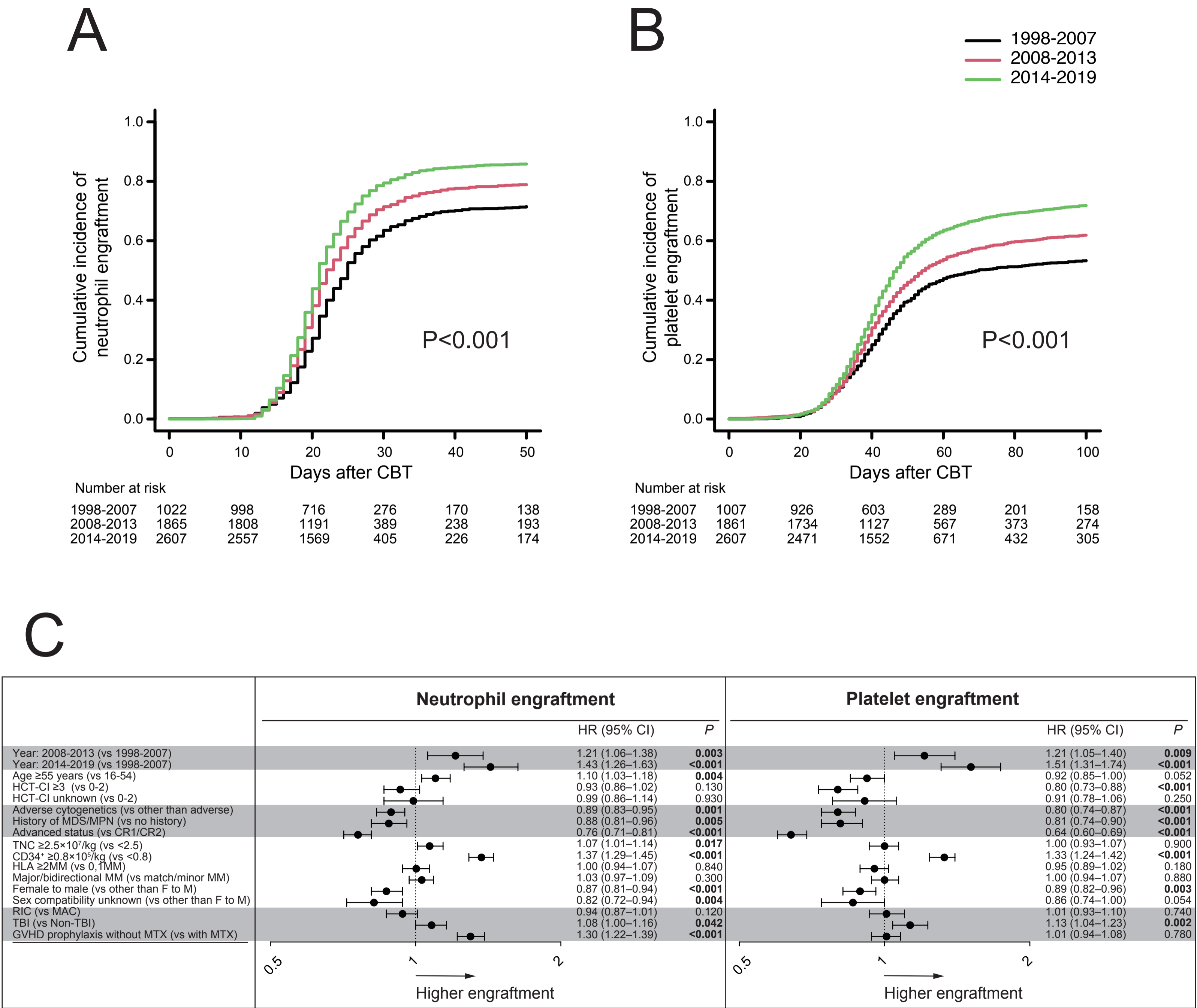

Supplement: Supplementary file 1 — Supplementary Figure 1 [file 41408_2022_678_MOESM1_ESM.pdf]
