# Supplementary material for: Improved trends in survival and engraftment after single cord blood transplantation for adult acute myeloid leukemia
Source: Blood Cancer J. 2022 May 25;12(5):81. doi: 10.1038/s41408-022-00678-6 (PMC9132934; doi:10.1038/s41408-022-00678-6)
Supplement: Supplementary file 2 — Supplementary Figure 2 [file 41408_2022_678_MOESM2_ESM.pdf]

Figure S2

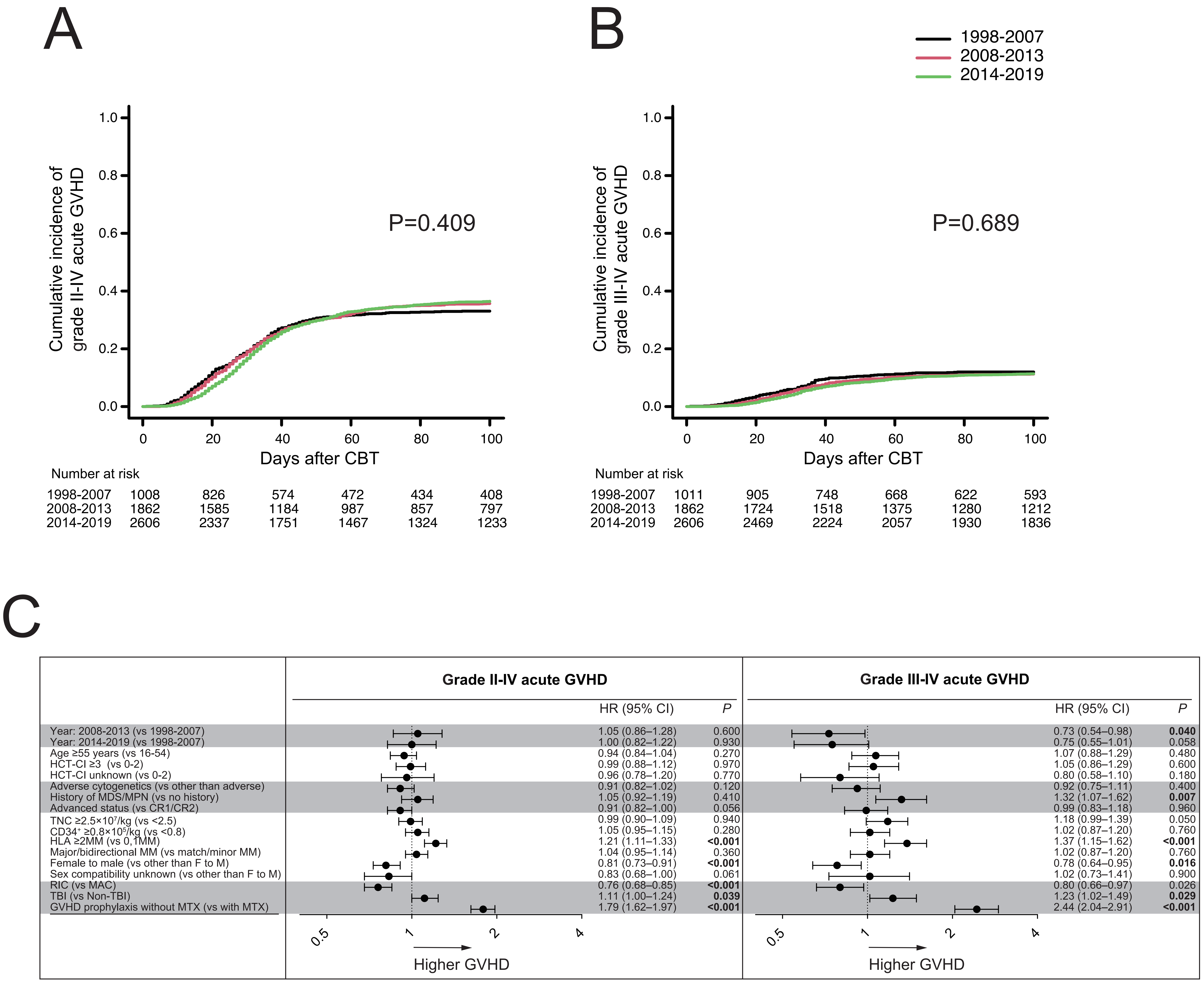

Supplementary Figure 2. The cumulative incidences of grade II-IV acute GVHD (A) and grade III-IV acute GVHD (B) after CBT according to the three time periods in the entire cohort. Forest plots for the adjusted hazard ratios and 95% confidence intervals of grade II-IV acute GVHD and grade III-IV acute GVHD in the multivariate analysis (C).
