# Supplementary material for: Improved trends in survival and engraftment after single cord blood transplantation for adult acute myeloid leukemia
Source: Blood Cancer J. 2022 May 25;12(5):81. doi: 10.1038/s41408-022-00678-6 (PMC9132934; doi:10.1038/s41408-022-00678-6)
Supplement: Supplementary file 3 — Supplementary Figure 3 [file 41408_2022_678_MOESM3_ESM.pdf]

Figure S3

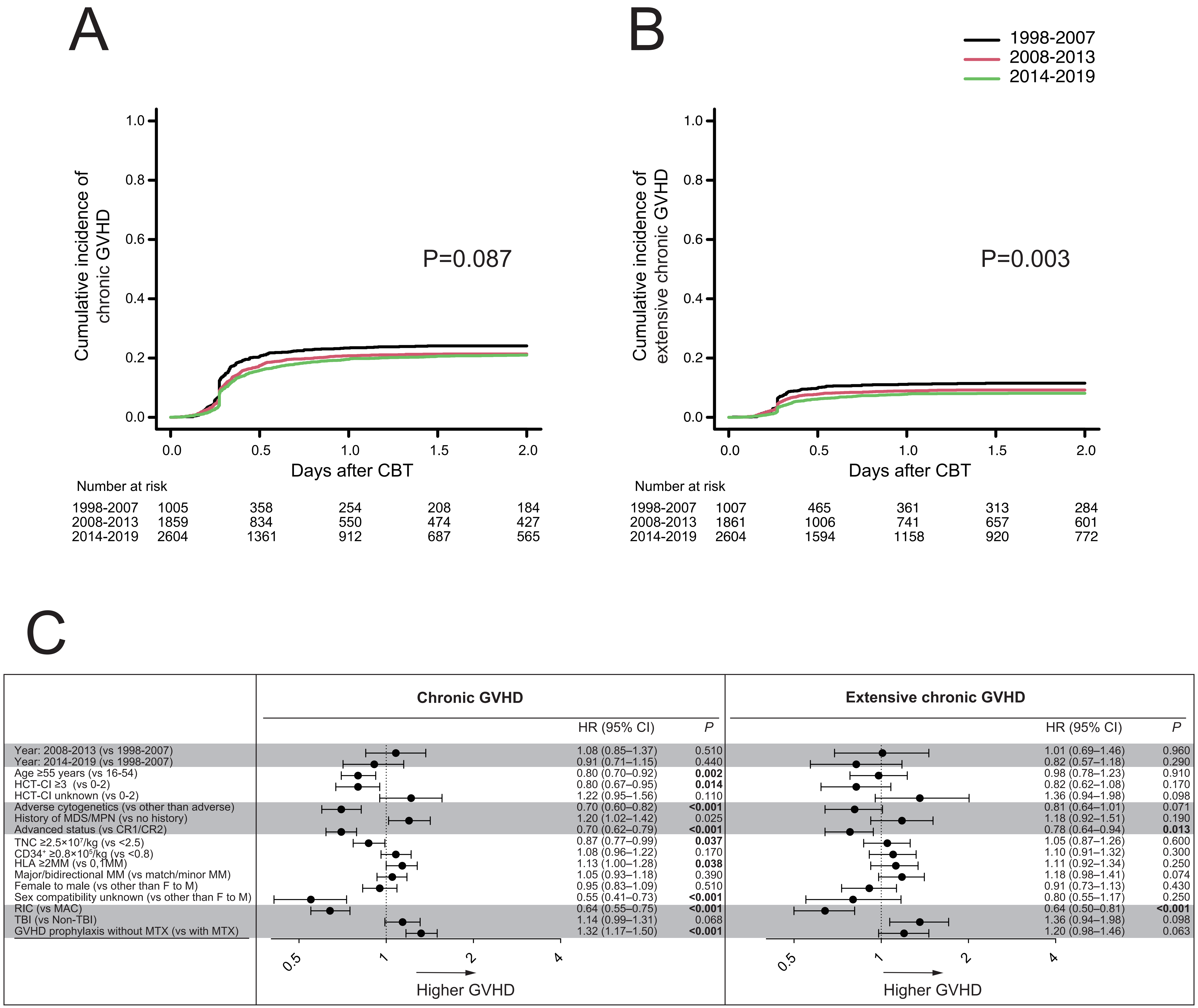

	Chronic GVHD HR (95% CI)	*P*	Extensive chronic GVHD HR (95% CI)	*P*
Year: 2008-2013 (vs 1998-2007)	1.08 (0.85–1.37)	0.510	1.01 (0.69–1.46)	0.960
Year: 2014-2019 (vs 1998-2007)	0.91 (0.71–1.15)	0.440	0.82 (0.57–1.18)	0.290
Age ≥55 years (vs 16-54)	0.80 (0.70–0.92)	**0.002**	0.98 (0.78–1.23)	0.910
HCT-CI ≥3 (vs 0-2)	0.80 (0.67–0.95)	**0.014**	0.82 (0.62–1.08)	0.170
HCT-CI unknown (vs 0-2)	1.22 (0.95–1.56)	0.110	1.36 (0.94–1.98)	0.098
Adverse cytogenetics (vs other than adverse)	0.70 (0.60–0.82)	**<0.001**	0.81 (0.64–1.01)	0.071
History of MDS/MPN (vs no history)	1.20 (1.02–1.42)	0.025	1.18 (0.92–1.51)	0.190
Advanced status (vs CR1/CR2)	0.70 (0.62–0.79)	**<0.001**	0.78 (0.64–0.94)	**0.013**
TNC ≥2.5×10^7/kg (vs <2.5)	0.87 (0.77–0.99)	**0.037**	1.05 (0.87–1.26)	0.600
$CD34^+$ ≥0.8×10^5/kg (vs <0.8)	1.08 (0.96–1.22)	0.170	1.10 (0.91–1.32)	0.300
HLA ≥2MM (vs 0,1MM)	1.13 (1.00–1.28)	**0.038**	1.11 (0.92–1.34)	0.250
Major/bidirectional MM (vs match/minor MM)	1.05 (0.93–1.18)	0.390	1.18 (0.98–1.41)	0.074
Female to male (vs other than F to M)	0.95 (0.83–1.09)	0.510	0.91 (0.73–1.13)	0.430
Sex compatibility unknown (vs other than F to M)	0.55 (0.41–0.73)	**<0.001**	0.80 (0.55–1.17)	0.250
RIC (vs MAC)	0.64 (0.55–0.75)	**<0.001**	0.64 (0.50–0.81)	**<0.001**
TBI (vs Non-TBI)	1.14 (0.99–1.31)	0.068	1.36 (0.94–1.98)	0.098
GVHD prophylaxis without MTX (vs with MTX)	1.32 (1.17–1.50)	**<0.001**	1.20 (0.98–1.46)	0.063

Supplementary Figure 3. The cumulative incidences of chronic GVHD (A) and extensive chronic GVHD (B) after CBT according to the three time periods in the entire cohort. Forest plots for the adjusted hazard ratios and 95% confidence intervals of chronic GVHD and extensive chronic GVHD in the multivariate analysis (C).
